# High BMI as a Risk Factor for Venous Thromboembolism in Middle-Aged and Older Patients Undergoing Isolated Medial Meniscus Repair: A Retrospective Cross-Sectional Study

**DOI:** 10.7759/cureus.90190

**Published:** 2025-08-15

**Authors:** Masanori Tamura, Nozomu Tsuji, Kazuhisa Sugiu, Yuki Okazaki, Takayuki Furumatsu

**Affiliations:** 1 Department of Orthopedic Surgery, Okayama University Hospital, Okayama, JPN; 2 Department of Orthopedics, Tsuyama Chuo Hospital, Tsuyama, JPN; 3 Department of Orthopedic Surgery, Japanese Red Cross Okayama Hospital, Okayama, JPN; 4 Department of Orthopedic Surgery, Okayama University Graduate School of Medicine, Dentistry, and Pharmaceutical Sciences, Okayama, JPN

**Keywords:** arthroscopy, case series, medial meniscus, repair, venous thromboembolism

## Abstract

Background and objective

Few studies have specifically investigated the incidence of venous thromboembolism (VTE) following arthroscopic meniscal repair in middle-aged or older patients. This study aimed to evaluate the prevalence of symptomatic VTE (sVTE) and identify its risk factors after medial meniscus repair (MMR).

Methods

Data were retrieved for 135 consecutive patients (male/female, 23/112; mean age, 66.3 years) who underwent arthroscopic MMR at a single center between March and October 2024. Fisher’s exact test was used to assess potential associations between categorical risk factors and sVTE.

Results

sVTE occurred in three patients (2.2%). Two required temporary percutaneous cardiopulmonary support, and one was treated with an inferior vena cava filter. A BMI ≥29.5 was identified as a significant risk factor, with an OR of 15.6 (95% CI: 1.33-182.6; p = 0.04).

Conclusions

Although arthroscopic meniscal surgery is generally considered safe, potential risk factors for thrombotic events should be carefully evaluated. Further research is warranted to determine the optimal thromboprophylaxis regimen following meniscus repair.

## Introduction

In recent years, guided by the principle of “Save the Meniscus,” the frequency of repair surgeries for isolated meniscal injuries, particularly medial meniscus posterior root tears, has increased, even among middle-aged and older patients [1-3]. One potential complication of surgery is the development of venous thromboembolism (VTE). The reported incidence of VTE after orthopedic surgery may depend on whether symptomatic VTE (sVTE) is included [4,5]. sVTE is rare after meniscal surgery, with an incidence of less than 1% following meniscectomy [6,7]. However, the non-weight-bearing period that may follow meniscus repair could increase the risk of VTE. Early weight bearing is often restricted for up to six weeks postoperatively, especially in cases of meniscus tears that disrupt the hoop function, such as root tears [8-10].

Previous reports have indicated that the incidence of sVTE after knee arthroscopy ranges from less than 1% to 10.9% [5,11-13]. The current guidelines of the American College of Chest Physicians do not recommend thromboprophylaxis for patients undergoing knee arthroscopy without a history of VTE [14]. Despite this, the 2022 International Consensus Meeting (ICM)-VTE emphasized the need for prophylaxis against VTE after knee arthroscopy if an unloading period is required. Therefore, thromboprophylaxis after arthroscopy remains controversial [15]. Although routine thromboprophylaxis is not recommended, an individualized approach based on risk factors has been suggested for patients at high risk. Risk factors for VTE in general orthopedic surgery include advanced age (>50 years, with risk increasing every additional 10 years), high BMI (>25), and a history of deep venous thrombosis (DVT) [2,15-17].

The purpose of the present study was to identify risk factors for postoperative sVTE following arthroscopic medial meniscus repair (MMR). We hypothesized that a high BMI may be a risk factor for the development of sVTE.

## Materials and methods

Patient recruitment and ethical approval

This retrospective study was approved by the Institutional Review Board of Okayama University Hospital (Rin1857). Given the retrospective nature of the study, the requirement for obtaining informed consent was waived. Patients who underwent arthroscopic MMR at a single institution between March and October 2024 were eligible for inclusion, and their data were reviewed retrospectively.

**Figure 1:**
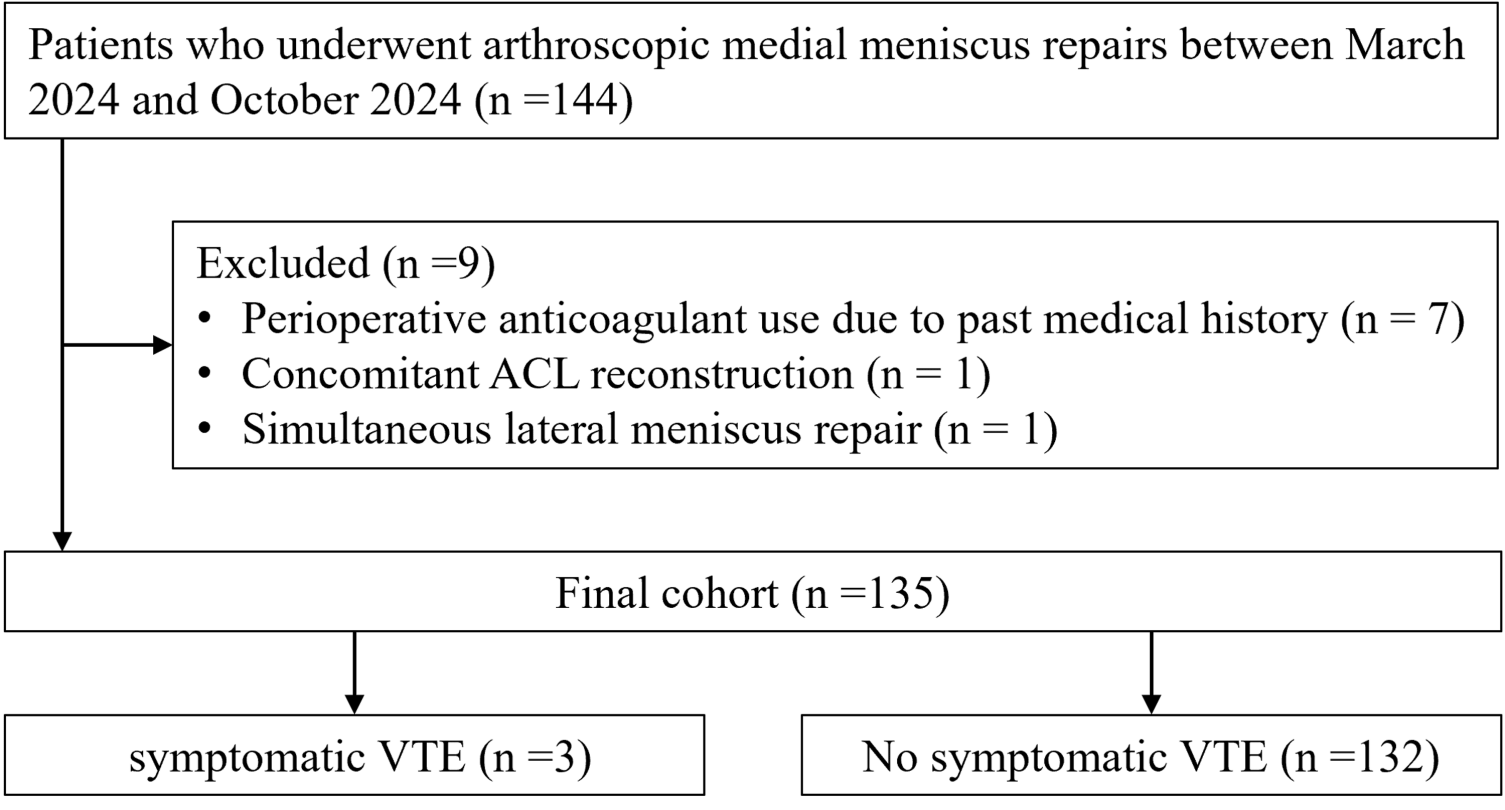
Flowchart presenting the study protocol ACL, anterior cruciate ligament; VTE, venous thromboembolism

The exclusion criteria were as follows: (1) medical history of perioperative thromboprophylaxis; (2) simultaneous reconstruction of the anterior cruciate ligament; and (3) simultaneous lateral meniscus suture repair (Figure 1).

Patients who met the inclusion criteria were classified according to the presence or absence of postoperative sVTE. Patient demographics (sex, affected side, age, and BMI) were assessed and compared between the groups.

Routine surgery and perioperative care

Arthroscopic meniscus repairs were performed under general anesthesia with the patient in the supine position. A femoral nerve block was administered under ultrasound guidance when deemed necessary by the anesthesiologist, using 20 mL of 0.375% ropivacaine in most cases. A tourniquet was applied during surgery.

Surgical techniques included pullout repair for root tears in 126 knees, inside-out repair for complex or longitudinal tears in five knees, and all-inside repair for horizontal tears in four knees. The inside-out technique was performed using 2-0 polyester sutures (FiberWire; Arthrex, Inc., Naples, FL, USA), while all-inside repairs were conducted using the FasT-Fix meniscal repair device (Smith & Nephew, Inc., Andover, MA, USA). For transtibial pullout repairs, 1.4-mm Minitape (Smith & Nephew) was used to place two simple stitches, and tibial fixation was achieved using a Biosure RG interference screw (Smith & Nephew).

As part of the routine thromboprophylaxis protocol at our facility, patients were encouraged to perform active ankle motion exercises while wearing postoperative elastic stockings. None of the patients received postoperative pharmacological thromboprophylaxis.

All patients were hospitalized until they could walk independently and were followed up once a month for three months postoperatively. According to the postoperative rehabilitation protocol, range-of-motion exercises and partial weight bearing were initiated on the first day after surgery. Range-of-motion exercises began with 30° of knee flexion, and the angle was increased by 30° each week until reaching 120°. Partial weight bearing of less than 25 kg was permitted on the first postoperative day, with the load increased by 25 kg each week until full weight bearing was achieved according to the patient’s body weight.

If any patient exhibited clinical signs or symptoms of VTE (e.g., abnormal vital signs, thoracic or lower limb pain, or swelling), contrast-enhanced CT and lower limb venous ultrasound were performed.

Statistical analysis

Statistical analyses were performed using EZR software (Saitama Medical Center, Jichi Medical University, Saitama, Japan). Given the low incidence of sVTE after MMR, Fisher’s exact test was used to evaluate associations between categorical risk factors and the occurrence of sVTE. Statistical significance was set at p < 0.05. An arbitrary cutoff value was determined for the continuous variable (BMI). ORs were calculated with 95% CIs.

## Results

A total of 144 patients were initially screened for eligibility. Nine were excluded: seven due to perioperative anticoagulant use based on medical history, one due to concomitant reconstruction of the anterior cruciate ligament, and one due to simultaneous lateral meniscus repair. Ultimately, 135 patients were enrolled in the study. The clinical demographics of the patients are summarized in Table 1.

**Table 1:** Clinical characteristics of the patients Values are presented as mean ± SD or as number (%).

Characteristic	Value
Sex, male/female	23/112
Age, years	66.3 ± 9.9
Age over 40, n (%)	134 (99)
Height, m	1.57 ± 0.10
Weight, kg	61.7 ± 13.5
BMI, kg/m²	24.9 ± 4.3
Affected side, right/left	65/70
General anesthesia, n (%)	135 (100)
Femoral nerve block, n (%)	75 (55.6)

**Figure 2:**
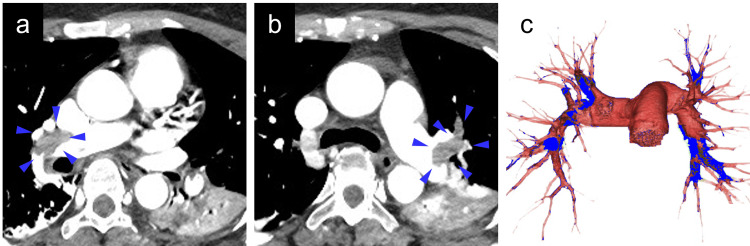
Contrast-enhanced CT (a, b) and 3D CT (c) showing massive PEs at eight days post-operation (a) PE in the segmental branches of the right pulmonary arteries (blue arrowheads). (b) PE in the main and segmental branches of the left pulmonary artery (blue arrowheads). (c) Multiple bilateral PEs (blue area) in the pulmonary artery (red area). PE, pulmonary embolism

**Figure 3:**
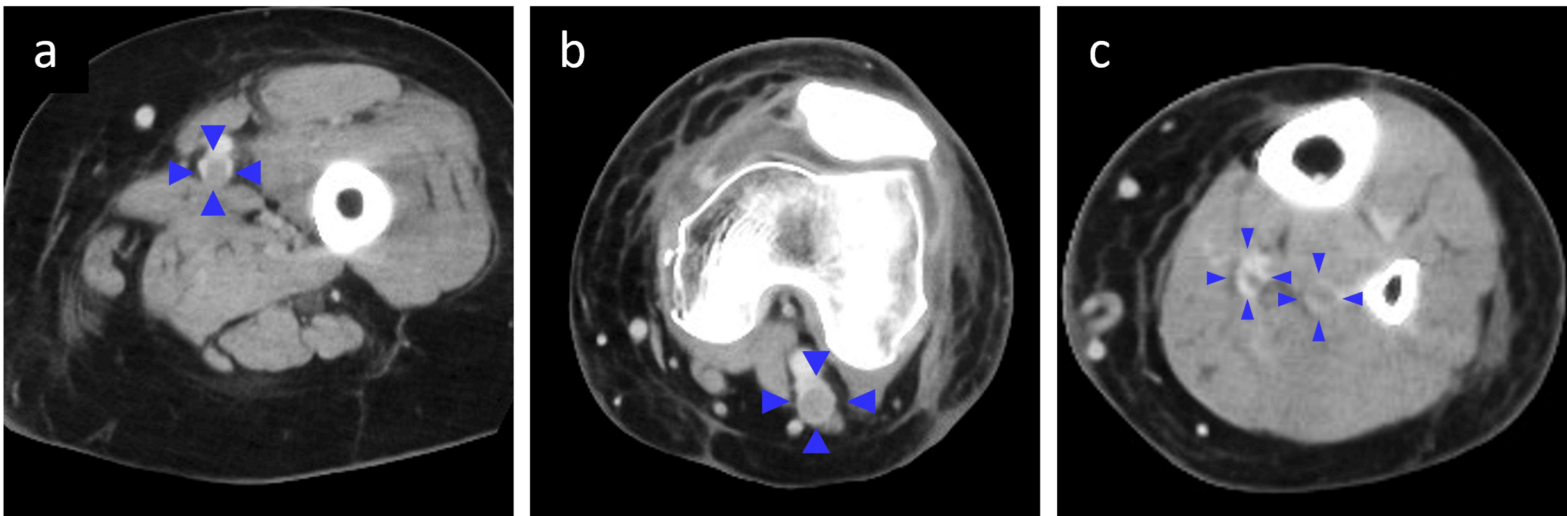
Contrast-enhanced CT axial views of the left leg with VTE at seven days post-operation (a) Femoral vein (enclosed by blue arrowheads). (b) Popliteal vein (enclosed by blue arrowheads). (c) Soleal veins (enclosed by blue arrowheads). VTE, venous thromboembolism

Three patients (2.2%) developed sVTE. Two of these patients had symptomatic pulmonary embolism (PE) requiring temporary percutaneous cardiopulmonary support (Figure 2). The remaining patient presented with a lower-extremity thrombus extending from the proximal femoral vein to the distal veins, necessitating placement of an inferior vena cava filter (Figure 3). All patients were diagnosed with sVTE within one week postoperatively. Oral medications were continued for three months postoperatively, and none of the patients reported symptoms related to PE or DVT.

A simple comparison of the demographic characteristics of patients in the sVTE and non-sVTE groups revealed no significant differences (Table 2).

**Table 2:** Comparison of patient demographics and clinical characteristics between groups Values are presented as the mean ± SD or number. p-Values for sex and affected side were calculated using FET. p-Values for age and BMI were calculated using WRS. FET, Fisher’s exact test; sVTE, symptomatic venous thromboembolism; WRS, Wilcoxon rank sum test

Characteristic	sVTE (n = 3)	No sVTE (n = 132)	Statistic (FET)	Statistic (WRS)	p-value (FET or WRS)
Sex, male/female	0/3	23/109	N/A	-	1
Affected side, right/left	0/3	65/67	N/A	-	0.25
Age, years	59.0 ± 15.6	66.5 ± 9.8	-	258.5	0.37
BMI, kg/m²	28.6 ± 4.5	24.8 ± 4.3	-	98.5	0.14

Upon analyzing various risk factors among patients with and without sVTE, a BMI ≥ 29.5 was identified as an independent risk factor, with an OR of 15.6 (95% CI: 1.33-182.6, p = 0.04; Table 3).

**Table 3:** Analysis of risk factors in patients with and without sVTE ^*^ p < 0.05 p-Value was calculated using FET. FET, Fisher’s exact test; sVTE, symptomatic venous thromboembolism

Characteristic	sVTE (n = 3)	No sVTE (n = 132)	Statistic (FET)	p-value (FET)	OR (95% CI)
BMI ≥ 29.5	2	15	N/A	0.04^*^	15.6 (1.33-182.6)
BMI < 29.5	1	117			

## Discussion

A key finding of this study was that BMI ≥ 29.5 kg/m² was a significant risk factor for the development of sVTE. Various reports have suggested that older age and high BMI increase the risk of thrombosis [15-17]. In the present study, BMI was identified as an independent factor, consistent with previous findings. In contrast, age was not an independent factor. Two of the three patients in the sVTE group were women in their 50s. This may be explained by the relationship between age and BMI, which are not strictly proportional; the average BMI decreases after age 70, and a negative correlation between age and BMI was reported in an epidemiological survey of meniscal tears [2,18]. Therefore, relatively younger patients do not necessarily have a low risk of sVTE after meniscal repair. Recently, Carroll et al. reported a high incidence of symptomatic deep vein thrombosis (10.7%) and PE (3.6%) after meniscal repair in patients over 40 years of age [19]. In their study, DVT was diagnosed at an average of 43 days postoperatively, which is relatively late. In contrast, in our study, all cases were diagnosed within two weeks after surgery. According to our postoperative protocol, patients with an average body weight of 62 kg are allowed full weight bearing by two weeks postoperatively. We believe that an early weight-bearing protocol may help reduce the risk of DVT.

Although no statistically significant differences were observed, all patients in the sVTE group were female, underwent left-sided surgery, and received general anesthesia. Some reports suggest that the incidence of VTE is similar or slightly higher in women than in men, particularly during the menopausal phase due to hormonal fluctuations; however, the differences are not statistically significant and remain debatable [20,21]. Regarding left-right differences in thrombosis, the left common iliac vein is anatomically compressed by the right common iliac artery, increasing the likelihood of left-sided thrombotic events. In a cohort study of patients with DVT, a higher incidence of thrombosis was reported in the left lower extremity compared with the right (60% vs. 40%) [22,23]. Regarding the type of anesthesia, recent reports have indicated that spinal anesthesia may reduce the risk of VTE after lower limb joint arthroplasty, possibly due to sympathetic blockade and reduced hypercoagulability [24]. This suggests that the choice of anesthesia may also be an important consideration in meniscus repair surgery. Although these factors were not identified as definitive risk factors for sVTE in our study, their combination may potentially contribute to increased risk. Further large-scale studies are warranted to investigate and identify high-risk patients.

Limitations

This study has several limitations. First, the retrospective design inherently carries the risk of selection bias and the potential for incomplete or inaccurate data collection. Second, the number of patients who developed sVTE was very small (n = 3), limiting the statistical power of our analysis. This also resulted in wide CIs for the OR, which may affect the reliability of the identified risk factor. Third, asymptomatic VTE cases were not evaluated. As only patients with clinical symptoms underwent diagnostic imaging, the true incidence of VTE may have been underestimated. Fourth, although all patients underwent similar surgical procedures and postoperative rehabilitation protocols, variations in anesthetic management and perioperative care could not be fully standardized and may have influenced the outcomes. Fifth, none of the three patients who developed sVTE had other potential risk factors, such as a history of cancer, congestive heart failure, chronic kidney disease, hormone replacement therapy, or the use of antiplatelet medications; however, a comprehensive evaluation of all patients for potential risk factors was not performed. Finally, this was a single-center study conducted in Japan, and the findings may not be directly applicable to other ethnicities or healthcare systems. Further multicenter, prospective studies with larger sample sizes are needed to validate our findings and establish optimal thromboprophylaxis strategies after meniscus repair.

## Conclusions

In the present study, three patients (2.2%) developed sVTE following MMR. BMI ≥ 29.5 kg/m² was identified as an independent risk factor for sVTE. Although arthroscopic meniscal surgery is generally considered safe, it is important to review the risk factors for VTE before and after surgery, particularly in patients at elevated risk. Appropriate thromboprophylaxis should be considered and administered when indicated. Our findings may serve as a useful reference for the development of precautionary guidelines before meniscal surgery.
